# Lanthionine Synthetase C-Like 2 Modulates Immune Responses to Influenza Virus Infection

**DOI:** 10.3389/fimmu.2017.00178

**Published:** 2017-02-21

**Authors:** Andrew Leber, Josep Bassaganya-Riera, Nuria Tubau-Juni, Victoria Zoccoli-Rodriguez, Pinyi Lu, Victoria Godfrey, Shiv Kale, Raquel Hontecillas

**Affiliations:** ^1^Nutritional Immunology and Molecular Medicine Laboratory, Biocomplexity Institute, Virginia Tech, Blacksburg, VA, USA

**Keywords:** influenza virus, immunoregulation, LANCL2, IL-10, drug discovery, infection resolution

## Abstract

Broad-based, host-targeted therapeutics have the potential to ameliorate viral infections without inducing antiviral resistance. We identified lanthionine synthetase C-like 2 (LANCL2) as a new therapeutic target for immunoinflammatory diseases. To examine the therapeutic efficacy of oral NSC61610 administration on influenza, we infected C57BL/6 mice with influenza A H1N1pdm virus and evaluated influenza-related mortality, lung inflammatory profiles, and pulmonary histopathology. Oral treatment with NSC61610 ameliorates influenza virus infection by down-modulating pulmonary inflammation through the downregulation of TNF-α and MCP-1 and reduction in the infiltration of neutrophils. NSC61610 treatment increases IL10-producing CD8+ T cells and macrophages in the lungs during the resolution phase of disease. The loss of LANCL2 or neutralization of IL-10 in mice infected with influenza virus abrogates the ability of NSC61610 to accelerate recovery and induce IL-10-mediated regulatory responses. These studies validate that oral treatment with NSC61610 ameliorates morbidity and mortality and accelerates recovery during influenza virus infection through a mechanism mediated by activation of LANCL2 and subsequent induction of IL-10 responses by CD8+ T cells and macrophages in the lungs.

## Introduction

Seasonal influenza causes an estimated 200,000 hospitalizations and 25,000–35,000 deaths annually in the United States, afflicting mainly people older than 65 years of age (1). Aside from the seasonal flu, pandemic influenza originating from emerging strains can significantly change the disease dynamics. Influenza pandemics cause considerable disease, with associated mortality ranging from approximately 50 million deaths during the 1918 pandemic to 1 to 4 million deaths in 1957 and approximately 1 million deaths in 1968. In 2009, a novel H1N1 virus emerged and spread rapidly in humans, causing severe disease in susceptible populations and high numbers of respiratory- and cardiovascular-related deaths (2). Overall, seasonal flu is one of the most relevant infectious disease-related public health problems since it causes important economic losses ranging from 71 to 166 billion dollars during one influenza pandemic (3).

Current approaches for the prevention and treatment of influenza infections include vaccination and early administration of antiviral drugs (4). Typically, a minimum of 6 months are needed to develop a new vaccine, creating a lag period between the identification of a new strain and the administration of the first vaccine doses, in which the population is unprotected against the virus (5). The use of antiviral drugs is associated with the emergence of resistance. A promising therapeutic avenue is the modulation of the host response to the virus by targeting the immune system in the pulmonary mucosa and systemically to minimize viral pneumonia and improve survival rates (6). The excessive release of pro-inflammatory cytokines in the lungs often leads to a cytokine storm, which is a key contributor to lung immunopathology and disease severity. For example, mortality induced by the highly pathogenic H5N1 strain correlates with high levels of circulating cytokines and chemokines (7). Hence, the use of drugs that target the host response instead of directly targeting the virus or its cytopathic effects is receiving attention for the treatment of infections (8, 9). In contrast to vaccines, immune modulators and inflammation blockers provide efficacy independently of changes in viral antigenicity, are fast acting, and are relatively inexpensive (10), although they have not been approved as host-targeted therapeutics. Moreover, in contrast to antivirals, immune modulators do not cause resistance.

We have recently investigated the potential role of abscisic acid (ABA), as a ligand of lanthionine synthetase C-like 2 (LANCL2) leading to elevation of intracellular cAMP and activation of protein kinase A (PKA) (11). We used molecular modeling approaches to predict the binding of ABA to LANCL2 (12). *In vitro* studies have confirmed direct binding of ABA to LANCL2 (13). Moreover, oral administration of ABA as a pre- and post-exposure therapeutic upregulates LANCL2 expression in the lungs of influenza-infected mice, reduces influenza virus-related immunopathology, and accelerates recovery in infected mice (14). LANCL2 is widely expressed in specialized organs of the immune system, including blood, spleen, lymph node, and thymus. LANCL2 is expressed by T cells, macrophages, endothelial and epithelial cells, and dendritic cells suggesting its potential as a target for immunoregulation (15). Other members of the LANCL family of proteins include LANCL2 which has been shown to contribute to cellular homeostasis and nervous system disorders (16, 17).

In this study, we examined the feasibility of using LANCL2 ligands to induce immunoregulatory responses and ameliorate morbidity and mortality associated with influenza virus infection. PubChem compound 247228, a 3,3′-bis(benzimidazolyl) terephthalanilide (BTT), also known as NSC61610 was selected from the National Cancer Institute Diversity Set II by LANCL2 structure-based virtual screening and previously shown to induce immunomodulatory effects in mouse models of colitis (18, 19). Additionally, the safety and efficacy profiles of BT-11, a newly developed LANCL2 ligand, are excellent based on single and 14-day repeated-dose toxicology studies in rats and in four mouse models of inflammatory bowel disease (IBD) (20–23). Given the demonstrated efficacy of ABA in accelerating recovery in mouse models of influenza virus infection (14), in this study, we investigated the effects of NSC61610 in a mouse model of infection, elucidating its underlying immunoregulatory mechanisms, LANCL2 dependency, and cell specificity in the lungs.

## Materials and Methods

### Animal Procedures

Eight- to ten-week-old wild-type C57BL/6 mice and LANCL2 knockout mice on a C57BL/6 background were challenged intranasally with 350 pfu/mouse of Influenza A H1N1pdm strain. NSC61610-treated mice within this study received 20 mg/kg/day of NSC61610 orally by gavage. Formulations of NSC61610 treatment were prepared in PBS containing 25 mg 2-hydroxypropyl-beta-cyclodextrin (HPBCD) per mg NSC61610. Untreated mice received equal volume of sterile PBS containing HPBCD. NSC61610 was given from day 0 to day 12, daily in 24-hour intervals. Mice treated with oseltamivir phosphate were given 10 mg/kg/day in two doses separated by 12 h. All mice were weighed daily. Mice were housed at the animal facilities at Virginia Tech. Mice (*n* = 10–15 per group and time point) were sacrificed at 3, 7, or 12 days post-infection (dpi), and samples were collected for analysis of gene expression, immunophenotyping of infiltrating cells, and histopathological examination. IL-10 neutralization was conducted by intraperitoneal injection of anti-IL10 antibody (R&D Systems #MAB417); 100 μg/mouse was injected on the same day as NSC61610 treatment initiation. The initial dosage was followed with a 50-μg/mouse dose on days 6 and 9 post-infection.

All experimental procedures were approved by the Institutional Animal Care and Use Committee (IACUC) of Virginia Tech and met or exceeded requirements of the Public Health Service/National Institutes of Health and the Animal Welfare Act. The IACUC approval IDs for the study were 10-157-VBI and 14-007-VBI. Mice were monitored every 4 h post-infection and humanely euthanized.

### Histopathology

Lung samples were collected at 3, 7, or 12 days post-infection and fixed in 10% buffered formalin. Samples were stained with hematoxylin and eosin and lesions were graded 0–4 on the following categories: (1) epithelial necrosis, (2) perivascular cuffing, (3) leukocytic infiltration of the mucosa and submucosa of large airways, and (4) terminal airway infiltration. Tissue slides were examined in an Olympus microscope (Olympus America Inc., Dulles, VA, USA).

### Quantitative Real-time RT-PCR

Total RNA was isolated from lungs. Quantitative PCR was performed on the cDNA using Taq DNA polymerase (Invitrogen, Carlsbad, CA, USA) and using previously described conditions (24). Purified amplicons were used to optimize quantitative real-time RT-PCR conditions and to generate standard curves. Primer concentrations and annealing temperatures were optimized for the iCycler iQ system (Bio-Rad) for each set of primers using the system’s gradient protocol. cDNA concentrations for genes of interest were examined by RT-PCR using an iCycler IQ System and the iQ SYBR green supermix (Bio-Rad) (24). A standard curve was generated for each gene using 10-fold dilutions of purified amplicons starting at 5 pg of cDNA. In order to determine the number of products synthesized during the real-time PCR, a melting curve analysis was performed on each product. RT-PCR was used to measure the starting amount of nucleic acid of each unknown sample of cDNA on the same 96-well plate. Results are presented as starting quantity of target cDNA (picograms) per microgram of total RNA as previously described (24).

### Immunophenotyping of Immune Cells Infiltrating the Lungs of Mice

The whole left lobe was collected in 15 mL of 1× RPMI supplemented with FBS, HEPES, and calcium chloride and chopped into small pieces to facilitate the digestion. Lung digestion was performed by adding 300 U/mL of Collagenase and 50 U/mL of DNAse and incubated for 60–90 min at 37°C under agitation. Cell yield was measured in a particle counter (Beckman Coulter) after digestion and subsequent filtration through 100 µm strainers. Red blood cells were eliminated by hypotonic lysis, and cells were finally resuspended in 1 mL of PBS containing 5% serum and 0.09% sodium azide (FACS buffer). Cells were incubated with combinations of up to 9 antibodies to markers [CD3 (clone: 145-2C11), CD4 (clone: GK1.5), CD8 (clone: 53-6.7), CD11b (clone: M1/70), CD11c (clone: HL3), CD19 (clone: MB19-1), CD45 (clone: 30-F11), F4/80 (clone: BM8), Gr1 (clone: 1A8), Ly6C (clone: HK1.4), MHC-II (clone: M5/114.15.2), NK1.1 (clone: PK136), SiglecF (clone: E50-2440), CX3CR1 (clone: AHP566), CD64 (clone: X54-5/7.1), PD-1 (clone: J43), and IL-10 (clone: JES5-16E3)]. Antibodies were purchased from eBioscience with the exception of CD11b, CD64, and SiglecF (Becton Dickinson) and CX3CR1 (BioRad). Thirty thousand events were computed in a LSRII flow cytometer (Becton Dickinson). Hematopoietic cell phenotype analysis was performed in FACS diva with the following gating discrimination: (1) live cells based of FS versus SS, (2) doublet exclusion based on FSC versus FSW, and (3) selection of CD45+ events.

### Plaque Assay

MDCK cells were grown to confluency within six-well plates. Cells were washed of serum containing media prior to exposure. Serial dilutions of virus sample were made in serum-free growth media containing fraction V BSA. Cells were incubated with 1 mL of virus dilution for 1 h at 37°C. Supernatant was removed and cells were washed. Cells were overlayed with a MEM agar mixture and incubated for 72 h. Overlay was removed, and wells were stained with crystal violet. Lowest dilution with at least 50 plaques was counted.

### Expression and Purification of the Recombinant LANCL2 Proteins

Transformed BL21(DE3) *E. coli* cells were initially cultured in Luria-Bertani medium with 100 µg/mL ampicillin at 37°C 240 RPM until the culture reached an A_600_ of 0.3. GST-LANCL2 was expressed by adding 0.1 mM isopropyl-β-d-thiogalactopyranoside. Induced cells were incubated for 16 h at 20°C 170 RPM. Cells were harvested by centrifugation 45 min 1,559 RCF and lysed by sonication in 50 mM Tris–HCl, pH 8.0, 150 mM NaCl with 0.3 mM Tris(2-carboxyethyl)phosphine (TCEP). Post membrane disruption, lysates were centrifuged at 17,211 RPM for 20 min at 4°C. GST-LANCL2 fusion protein was purified by affinity chromatography using Glutathione (GSH)-Sepharose-4B (GE Healthcare). GST-LANCL2 was eluted from GSH-Sepharose-4B by incubating the resin with 10 mM GSH in 50 mM Tris–HCl, pH 8.0, 150 mM NaCl with 0.3 mM TCEP. GST-LANCL2 proteins were run through a gel filtration column. The fusion proteins were further purified by the AKTA Fast protein liquid chromatography purification systems (GE Healthcare). Protein concentrations were determined by bicinchoninic acid assay. Protein purity was assessed by SDS-PAGE; gels were stained with ProSieve Blue Protein Staining solution.

### Sensor Chip Preparation

Direct binding experiments were performed *via* the Biacore T200 surface plasmon resonance (SPR) Technology (Georgetown University). The flow rates were 10 µL/min for all capture and initial testing studies and 100 µL/min for affinity studies. The GST-LANCL2 was immobilized to a CM4 chip by amine coupling method. Two adjacent surfaces were activated by injection of a 1:1 (v:v) mixture of 0.1 M *N*-hydroxysuccinimide (NHS) and 0.4 M 1-ethyl-3-(3-dimethyl-aminopropyl)-carbodiimide hydrochloride (EDC) for 720 s. Experimental flow cell was then injected with the GST-LANCL2 that was diluted in 10 mM pH 5.5 sodium acetate buffer to a final concentration of 25 µg/mL. Both experimental and the reference flow cells were inactivated by injection of 1 M Ethanolamine–HCl pH 8.0 for 720 s.

### Kinetic Studies of ABA and NSC61610

A final GST-LANCL2 surface density (R_L_) of 7,500 RU equivalent to an R_max_ of 53 and 26 RU for NSC61610 and ABA, respectively, was obtained. Following initial binding studies, it was determined that binding of small molecules to the LANCL2 was occurring with a fast off rate. Therefore, no regeneration step was required following small molecule injections. Binding affinity was calculated following injection of small molecules at four different concentrations (12.5, 6.25, 3.13, and 1.57 µM) in triplicates. Injection time was 60 s and dissociation time was 300 s. Running buffer for binding studies was 25 mM MOPS (pH 6.5), 150 mM NaCl, 0.05% P-20, 5% DMSO. Data were analyzed by using the BiaEvaluation software v1 (GE Healthcare) with 1:1 binding model for steady state affinity. Raw data were exported and graphed using Prizm for Mac v5.0d.

### Statistical Analysis

Data from the first mouse challenge study were analyzed as a series of factorial arrangement designs. To determine the statistical significance of the model, we performed analysis of variance (ANOVA) using the general linear model procedure of Statistical Analysis Software, and *p*-value <0.05 was considered to be significant. When the model was significant, ANOVA was followed by Fisher’s protected least significant difference multiple comparison method.

## Results

### LANCL2 Aids in Resolution and Recovery from Influenza Infection

**Figure 1:**
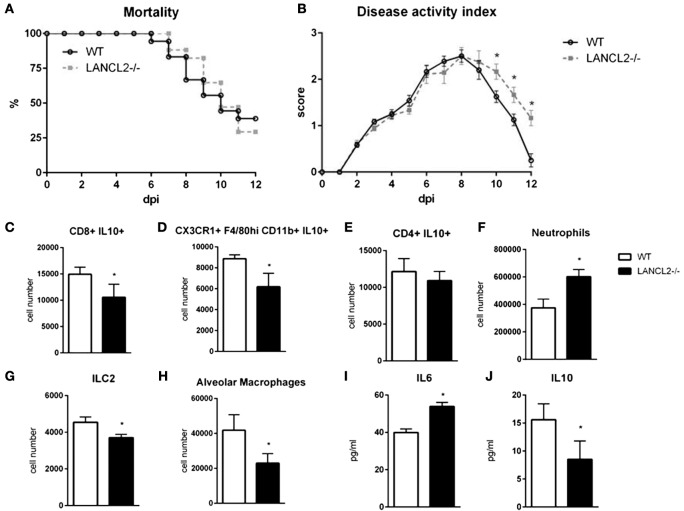
**Loss of LANCL2 impairs resolution of influenza virus infection**. Mortality **(A)** and disease activity **(B)** of WT and LANCL2−/− mice infected with influenza. Cell number of CD8+ IL10+ T cells **(C)**, IL10-producing macrophages **(D)**, CD4+ IL10+ T cells **(E)**, neutrophils **(F)**, type 2 innate lymphoid cells **(G)**, and alveolar macrophages **(H)** at day 12 post-infection by flow cytometry. Concentration of IL-6 **(I)** and IL-10 **(J)** in lung homogenate at day 12 post-infection by cytokine bead array. Data points and error bars represent mean ± standard error of the mean (SEM). Asterisks (*) denote statistically significant (*p* < 0.05) differences between the treatment group and control (*n* = 12).

To assess the role of LANCL2 in the response to influenza infection, wild-type C57BL6/J mice and LANCL2−/− mice were infected with 350 pfu/mouse of influenza A H1N1/California/04/09. Notably, LANCL2−/− mice had a decreased rate of survival and prolonged presence of symptoms (Figures 1A,B). Within the lungs of infected mice, LANCL2−/− possessed lower levels of IL-10 and higher levels of IL-6 at day 12 post-infection (Figures 1I,J). In general, LANCL2−/− displayed lower numbers of IL-10-producing cells within the lungs during the same period and specifically within CX3CR1+ macrophage and CD8+ T cell populations (Figures 1C–E). LANCL2−/− mice also showed lesser numbers of tissue repair and homeostasis cell types in alveolar macrophages and type 2 innate lymphoid cells at day 12 post-infection(Figures 1G,H) while displaying greater number of neutrophils at day 7 post-infection (Figure 1F).

### Myeloid LANCL2 Is Required for Modulation of Regulatory Responses

**Figure 2:**
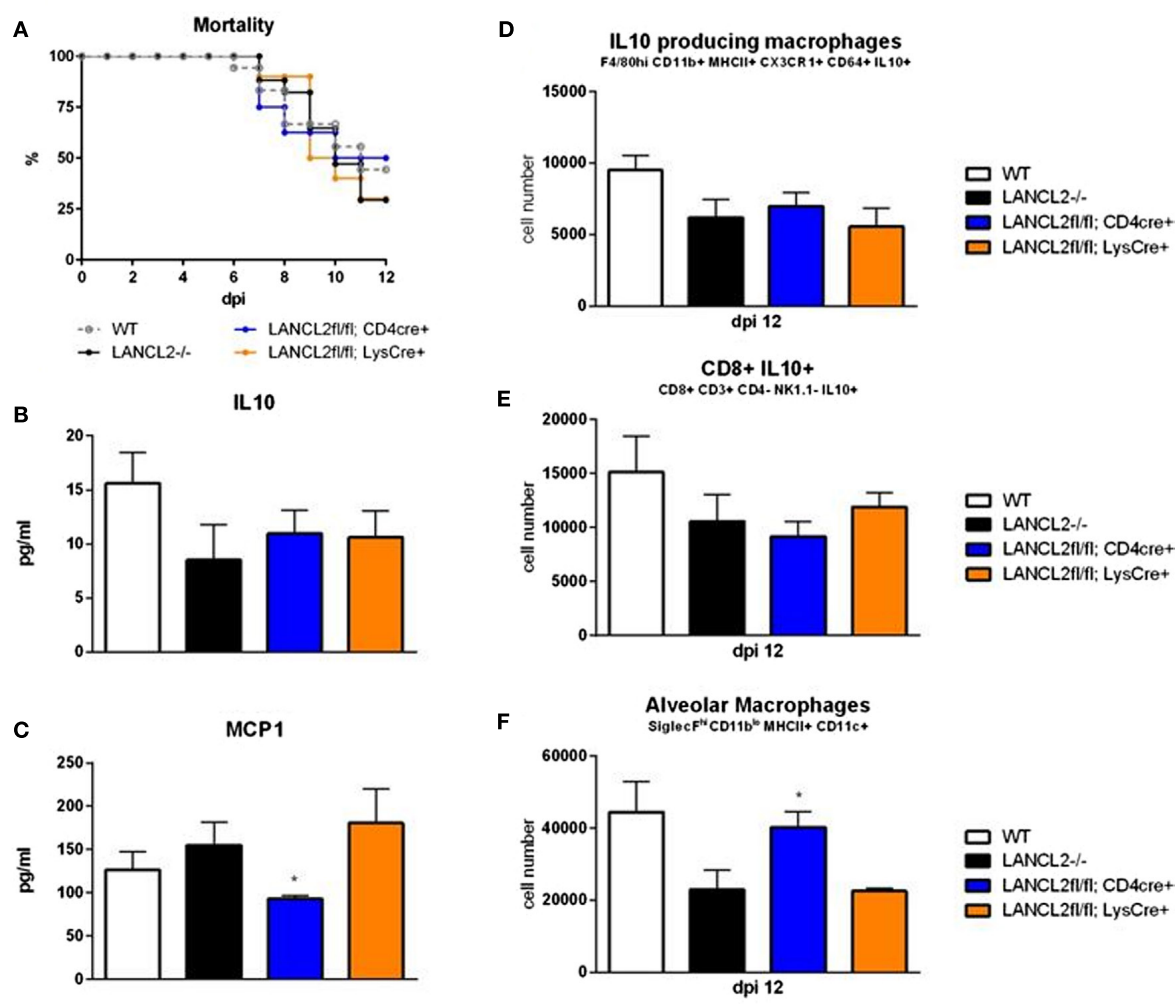
**Myeloid LANCL2 is required for modulation of regulatory responses**. Mortality **(A)** for LANCL2−/−, LANCL2fl/fl;CD4Cre+, and LANCL2fl/fl;LysCre+ mice infected with influenza H1N1. Concentration of IL-10 **(B)** and MCP1 **(C)** within lung homogenate by cytokine bead array. Cell number of CX3CR1+ IL-10+ macrophages **(D)**, CD8+ IL-10+ T cells **(E)**, and alveolar macrophages **(F)** in lungs by flow cytometry at 12 days post-infection. Data points and error bars represent mean ± SEM. Asterisks (*) denote statistically significant (*p* < 0.05) differences between genotypes (*n* = 8).

Using a cre-recombinase system, myeloid (LANCL2fl/fl;LysCre+) and T cell-(LANCL2fl/fl;CD4Cre+) specific knockouts of LANCL2 were generated. Myeloid cell knockouts recapitulated the LANCL2−/− phenotype in terms of mortality (Figure 2A). Both cell specific knockouts displayed decreased levels of IL-10 within the lungs at day 12 post-infection similar to the LANCL2−/− (Figure 2B). In addition, IL-10-producing macrophages and CD8+ T cells were similarly decreased in CD4Cre+ and LysCre+ compared to LANCL2−/− (Figures 2D,E). However, LANCL2fl/fl;CD4Cre+ mice had significantly increased numbers of alveolar macrophages compared to LANCL2−/− and LANCL2fl/fl;LysCre+ mice (Figure 2F). The number of alveolar macrophages in LANCL2fl/fl;CD4Cre+ was similar to numbers in untreated wild-type mice. LANCL2fl/fl;CD4Cre+ mice also displayed lower concentrations of MCP-1 in lung homogenate than LANCL2−/− and LANCL2fl/fl;LysCre+ mice (Figure 2C). Together, these data suggest that a myeloid-specific LANCL2 deficiency is capable of producing the same effects as the full body deletion of the protein.

### Oral NSC61610 Treatment Improves Influenza Virus-Associated Lung Immunopathology and Protects Mice against Lethal Influenza Virus Infection

**Figure 3:**
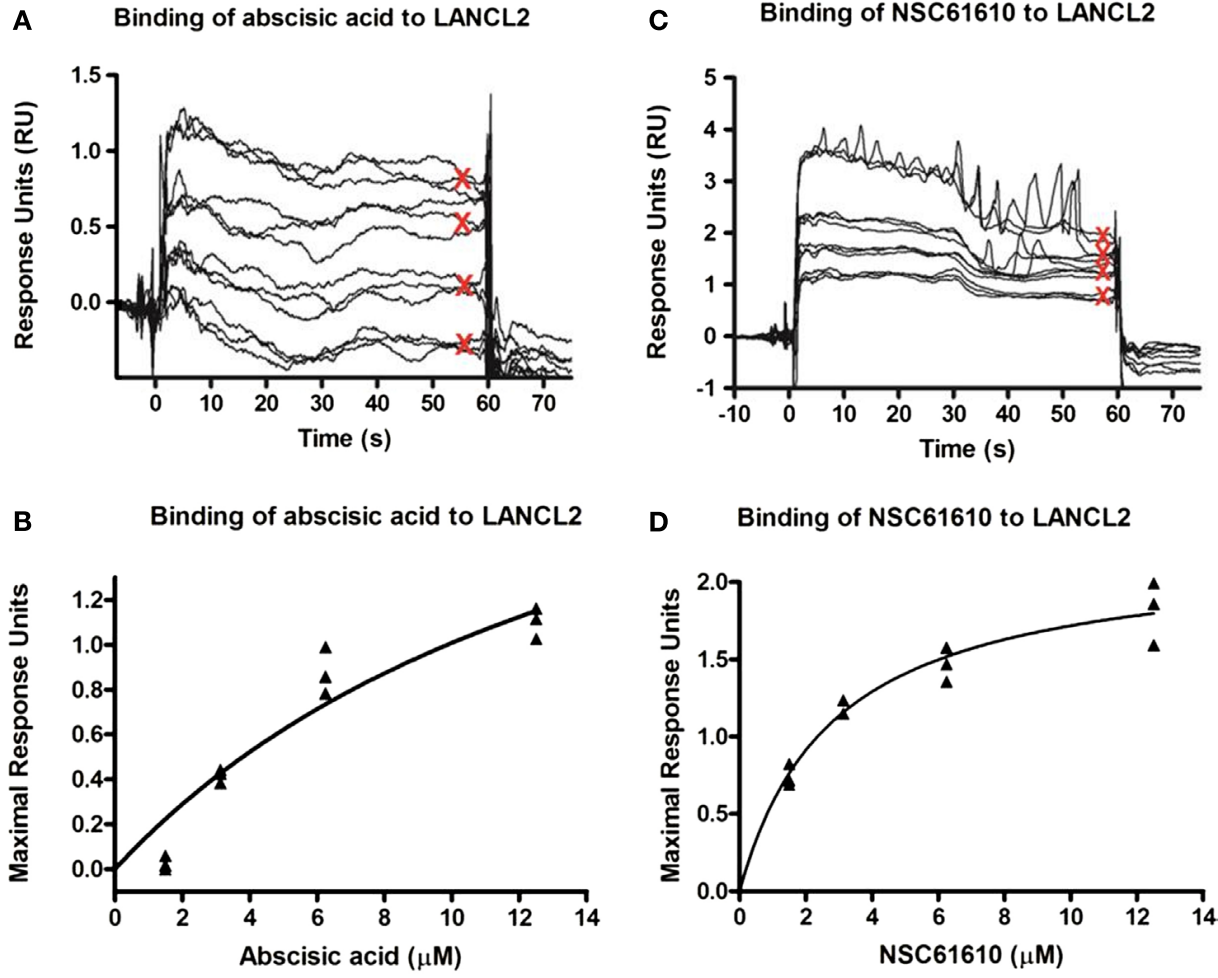
**Binding kinetics of lanthionine synthetase C-like 2 (LANCL2) with abscisic acid (ABA) and NSC61610**. Surface plasmon resonance (SPR) sensorgrams for the binding of varying concentrations of ABA (12.5, 6.25, 3.13, and 1.57 µM) to immobilized LANCL2 **(A)**. Plot of maximal resonance unit versus concentration of ABA **(B)**. Steady-state dissociation constant was calculated to be 2.252 µM utilizing a 1:1 binding model. SPR sensograms for the binding of varying concentrations of NSC61610 (12.5, 6.25, 3.13, and 1.57 µM) to immobilized LANCL2 **(C)**. Plot of maximal resonance unit versus concentration of NSC61610 **(D)**. Steady state dissociation constant was calculated to be 2.305 µM utilizing a 1:1 binding model.

**Figure 4:**
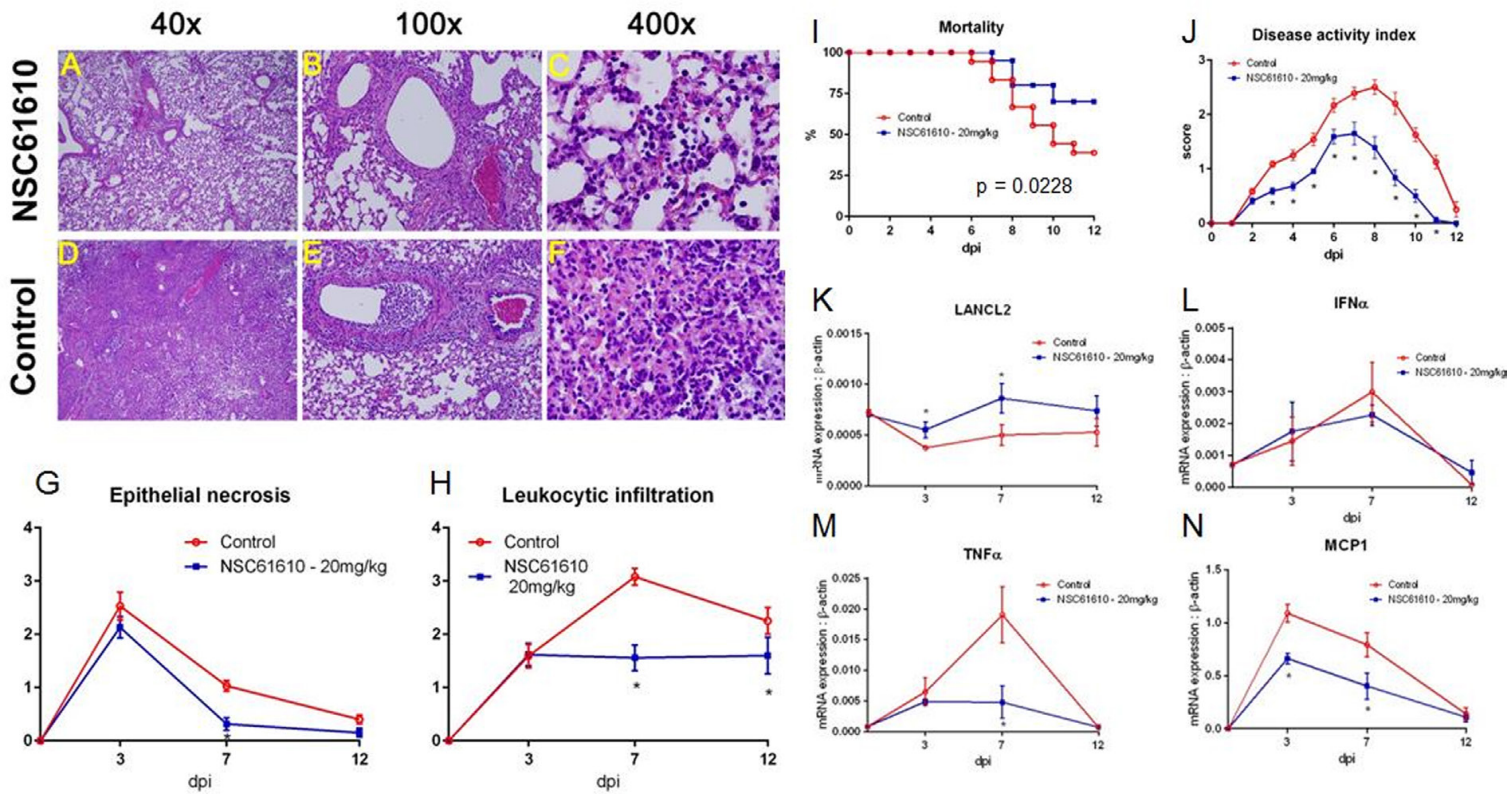
**NSC61610 treatment decreases severity and improves recovery from influenza virus infection**. Representative photomicrographs of H&E stained lung sections from NSC61610 **(A–C)** and control treated **(D–F)** mice following influenza infection. Summary of epithelial necrosis **(G)** and leukocytic infiltration **(H)** scores. Mortality **(I)** and disease activity **(J)** of WT mice infected with influenza with no treatment and NSC61610 treatment. mRNA expression of LANCL2 **(K)**, IFNα **(L)**, TNFα **(M)**, and MCP1 **(N)** at days 0, 3, 7, and 12 post-infection in lung tissue during influenza infection of wild-type mice administered PBS or NSC61610 (20 mg/kg) by qRT-PCR. Data points and error bars represent mean ± SEM. Asterisks (*) denote statistically significant (*p* < 0.05) differences between the treatment group and control (*n* = 12).

After observation of the importance of LANCL2 in the resolution phase of infection, we sought to identify if novel ligands of LANCL2 could aid in the response to influenza. First, to validate *in silico* predictions that NSC61610 would bind to LANCL2, LANCL2 was expressed in *E. coli*. The binding of NSC61610 to the purified protein was analyzed *via* SPR and compared to the natural ligand of LANCL2, ABA (Figure 3). Steady state equilibrium constants (*K*_D_) were determined to be 2.252 µM for the ABA–LANCL2 interaction, while the NSC61610–LANCL2 was 2.305 µM. Following the validation of binding, we performed an assessment of the ability of NSC61610 to improve influenza virus-induced morbidity and/or mortality. C57BL/6 wild-type mice were challenged with 350 pfu/mouse of influenza A H1N1/California/04/09. At 12 days post-infection, the mortality rate was 60% in the control group versus 30% in the NSC61610-treated mice (Figure 4I). The onset of mortality differed by 1 day with the untreated wild-type group beginning on day 6 compared to day 7 in the NSC61610-treated groups. Mice were also scored on a daily basis through observation of physical activity and appearance. NSC61610-treated mice were significantly more active and showed less signs of distress by this measure (Figure 4J). Our clinical data show that oral NSC61610 treatment improves the resolution of infection and accelerates the recovery from disease. These findings are in line with the lower mortality rates recorded in the group that received oral NSC61610 treatment.

To determine whether the improved clinical symptoms observed in NSC61610-treated mice were accompanied by decreased lung pathology, we evaluated microscopic lung lesions at 3, 7, and 12 days post-infection. Examination of lung tissue was based on epithelial necrosis, including presence of debris in large and intermediate size airways, and leukocytic infiltration of the mucosa and submucosa of large airways. Chronologically, the first pulmonary lesion detected was epithelial cell necrosis, with presence of necrotic cells in the airway compartment at day 7 (Figures 4B,E) and marginated leukocytes in adjacent blood vessels and in some cases with perivascular edema. At later stages, the predominant findings were leukocytic infiltration of the mucosa and submucosa of large and medium size airways (Figures 4A,B,D,E). This was followed by the presence of inflammatory cells in the terminal airways (Figures 4C,F) on day 7 post-infection. To determine whether NSC61610 ameliorated lung immune-pathology associated with infection, we scored these lesions from 0 to 4 depending on extent and severity. The analysis shows that NSC61610 indeed exerted a significant impact in the extent of lung epithelial necrosis (Figure 4G) and leukocytic infiltration (Figure 4H). The decline in epithelial necrosis scores occurred 2 days earlier in NSC61610-treated mice when compared to untreated mice. Additionally, the severity of leukocytic infiltration was significantly lower in NSC61610-treated mice on day 7 post-challenge than in untreated mice. In untreated mice, the immune cell infiltration increased to reach a maximum score of 3 on day 7. In the NSC61610-treated mice the score was maintained under 2 throughout the experiment, suggesting lower inflammatory cells recruited to the lungs.

In NSC61610-treated wild-type mice, oral treatment of NSC61610 increases expression of LANCL2 throughout the time course of influenza infection (Figure 4K). To confirm the decrease in inflammatory cell types during the peak of infection in NSC61610-treated mice, we measured the mRNA expression of inflammatory markers, IFNα, TNFα and MCP1, in the lungs of infected mice (Figures 4L–N). Notably, NSC61610 treatment reduced expression of TNFα, at day 7 post-infection, and MCP1 at days 3 and 7 post-infection. IFNα was not significantly altered at any of the observed time points.

### NSC61610 Promotes Immunological Mechanisms of Regulation and Repair in the Lungs

**Figure 5:**
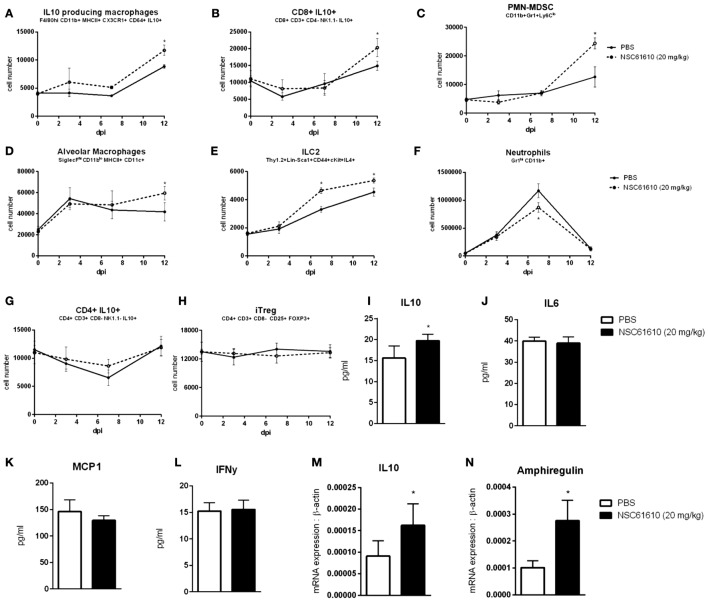
**NSC61610 Promotes Regulatory Responses during Resolution Phase of Infection**. Cell number of IL10-producing macrophages **(A)**, CD8+ IL10+ T cells **(B)**, polymorphonuclear myeloid-derived suppressor cells **(C)**, alveolar macrophages **(D)**, type 2 innate lymphoid cells **(E)**, neutrophils **(F)**, CD4+ IL10+ T cells **(G)**, and induced T regulatory cells **(H)** through day 12 post-infection by flow cytometry. Concentration of IL-10 **(I)**, IL-6 **(J)**, MCP-1 **(K)**, and IFNγ **(L)** in lung homogenate at day 12 post-infection by cytokine bead array. mRNA expression of IL10 **(M)** and amphiregulin **(N)** in lung at day 12 post-infection by qRT-PCR. Data points and error bars represent mean ± standard error of the mean (SEM). Asterisks (*) denote statistically significant (*p* < 0.05) differences between the treatment group and control (*n* = 12).

Oral administration of NSC61610 increases the number of IL-10-producing macrophages and CD8+ T cells (Figures 5A,B). The numbers of IL-10-producing CD4+ T cells and induced T regulatory cells were not changed by treatment (Figures 5G,H). Numbers of polymorphonuclear myeloid-derived suppressor cells, alveolar macrophages and innate lymphoid cell type 2 were also significantly increased at day 12 post-infection (Figures 5C–E). Meanwhile, neutrophils were slightly suppressed at the peak, day 7, of infection (Figure 5F). Using a cytokine bead array, the concentration in lung homogenate of cytokines was measured at day 12 post-infection. The concentration of IL-10 was significantly increased by oral NSC61610 treatment (Figure 5I). Trends in IL-10 production were further confirmed by qRT-PCR with lung tissue (Figure 5M). No significant trends were observed in IL-6, MCP1 or IFNγ at day 12 post-infection (Figures 5J–L). Expression of amphiregulin was also observed to be significantly increased by oral NSC61610 treatment (Figure 5N).

### LANCL2 Is Required for the Beneficial Effects of NSC61610

**Figure 6:**
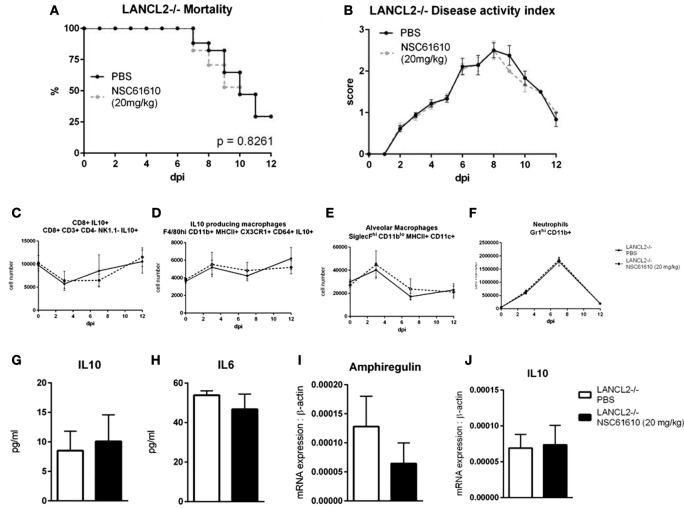
**Loss of LANCL2 impairs recovery and diminishes clinical effects of NSC61610 treatment**. Mortality **(A)** and disease activity **(B)** of LANCL2−/− mice infected with influenza with no treatment and NSC61610 treatment. Cell number of CD8+ IL10+ T cells **(C)**, IL10-producing macrophages **(D)**, alveolar macrophages **(E)**, and neutrophils **(F)** through day 12 post-infection by flow cytometry. Concentration of IL-10 **(G)** and IL-6 **(H)** in lung homogenate at day 12 post-infection by cytokine bead array. mRNA expression of amphiregulin **(I)** and IL10 **(J)** in lung at day 12 post-infection by qRT-PCR. Data points and error bars represent mean ± SEM. Asterisks (*) denote statistically significant (*p* < 0.05) differences between the treatment group and control (*n* = 12).

As NSC61610 is a predicted ligand of LANCL2, we sought to evaluate the specificity of the ligand’s effects in LANCL2−/− mice. To determine if the efficacy of NSC61610 treatment is dependent on the presence of LANCL2, we administered PBS or NSC61610 to infected LANCL2−/− mice. No significant differences were apparent in mortality or disease activity scores (Figures 6A,B). Additionally, no differences were observable in cellular (Figures 6C–F) or molecular (Figures 6G–J) measures noted to be changed in wild-type mice treated with NSC61610.

### Effects of NSC61610 Are Mediated by IL-10

**Figure 7:**
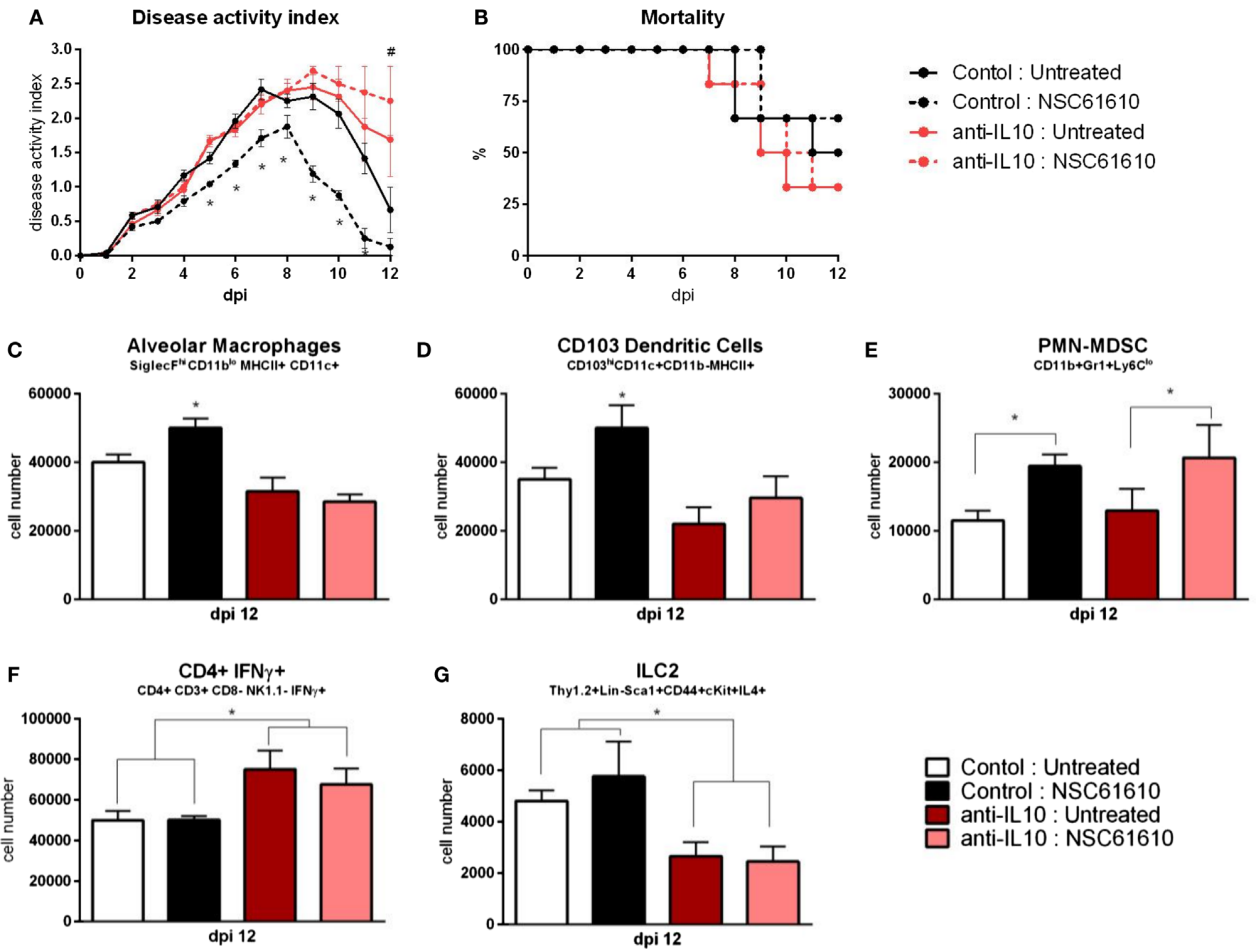
**IL10 neutralization abrogates efficacy of NSC61610**. Disease activity **(A)**, and mortality **(B)** of wild-type mice following IL10 neutralization during influenza infection. Cell number of alveolar macrophages **(C)**, CD103+ dendritic cells **(D)**, polymorphonuclear myeloid-derived suppressor cells **(E)**, CD4+ IFNγ+ T cells **(F)**, and type 2 innate lymphoid cells **(G)** within lungs at day 12 post-infection by flow cytometry. Data points and error bars represent mean ± SEM. Asterisks (*) denote statistically significant (*p* < 0.05) differences between the NSC61610 treatment group and control (*n* = 8). Number signs (#) denote statistically significant (*p* < 0.05) differences between neutralization treatment group and control (*n* = 8).

Mice were infected with influenza virus as previously. After infection, mice were administered an IL-10 neutralizing antibody or isotype control. IL-10-neutralized mice treated with NSC61610 exhibited no differences from untreated IL-10-neutralized mice in clinical measures of disease activity index and mortality (Figures 7A,B). The isotype control antibody did not dampen the efficacy of NSC61610 against influenza. The IL-10 neutralization inhibited the increases in alveolar macrophages, CD103+ dendritic cells, and ILC2 experienced with NSC61610 treatment (Figures 7C,D,G). The neutralization of IL-10 also significantly increased the number of IFNγ-producing CD4+ T cells (Figure 7F). However, the neutralization of IL-10 did not reduce the NSC61610-mediated increase in myeloid-derived suppressor cells (Figure 7E).

### NSC61610 or Combined Therapy with NSC61610 and Tamiflu Outperforms Tamiflu Alone

**Figure 8:**
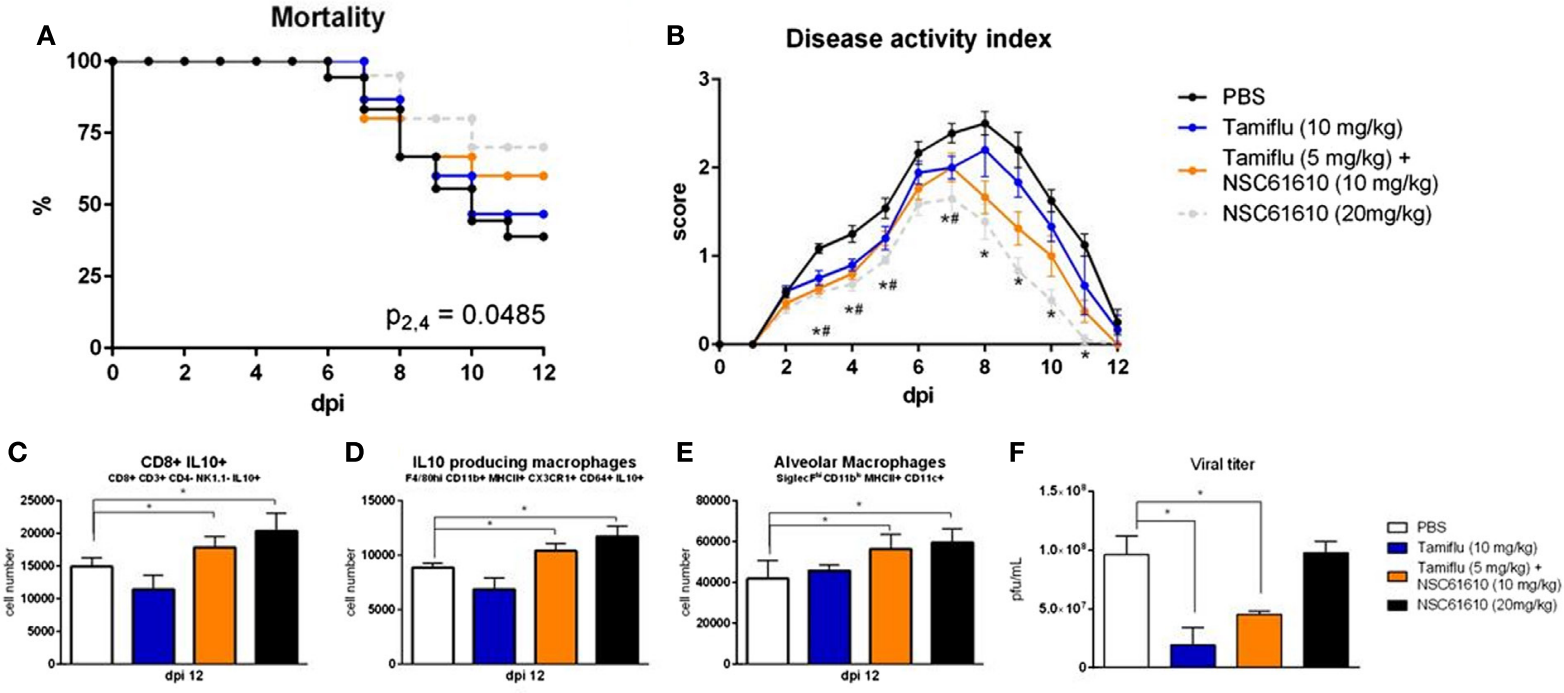
**Combination of NSC61610 and Tamiflu promotes regulatory responses and suppresses viral replication**. Mortality **(A)** and disease activity **(B)** of mice treated with PBS, Tamiflu (10 mg/kg/day), Tamiflu and NSC61610 (5 and 10 mg/kg/day respectively), or NSC61610 alone (20 mg/kg/day). Cell number of CD8+ IL10+ T cells **(C)**, IL10-producing macrophages **(D)**, and alveolar macrophages **(E)** at day 12 post-infection by flow cytometry. Viral titer **(F)** within the lungs on day 3 post-infection by MDCK cell plaque assay. Data points and error bars represent mean ± SEM. Asterisks (*) denote statistically significant (*p* < 0.05) differences between the combination treatment group and control (*n* = 12). Number signs (#) denote statistically significant (*p* < 0.05) differences between Tamiflu treated and control groups (*n* = 12). Ampersands (&) denote statistically significant (*p* < 0.05) differences between combination and single treatment groups (*n* = 12).

To determine whether the treatment efficacy of NSC61610 could be further increased by combination with an antiviral agent, we administered NSC61610 in combination with oseltamivir phosphate, an active ingredient of Tamiflu. NSC61610 and combination treatment improved the overall mortality and disease activity compared with treatment with oseltamivir alone (Figures 8A,B). Notably, treatment only with Tamiflu did not promote cellular regulatory responses within the lungs while the combination therapy retained the regulatory benefits of NSC61610 treatment (Figures 8C–E). After the observation that treatment with NSC61610 induces regulatory and anti-inflammatory effects, we sought to determine if these effects impacted the viral burden within the lungs. The amount of virus was titrated by plaque assay of MDCK cells. Treatment with NSC61610 did not alter the amount of virus detected. Both Tamiflu and combination therapies significantly reduced the viral titer (Figure 8F).

## Discussion

The LANCL2 pathway has emerged as a therapeutic target for inflammatory, chronic, and immune-mediated diseases (15). By using pharmacologic activation of LANCL2 and loss-of-function approaches in knockout mice, we validate the LANCL2 pathway as a putative target for the treatment of influenza infection. ABA binds to LANCL2 (13) and causes elevation of intracellular cAMP and activation of PKA in macrophages (11). In addition, ABA suppressed LPS-induced inflammation in mice (11), experimental colitis (25, 26), and accelerated recovery in influenza virus-driven lung immunopathology (14). Previous work on ABA illustrates the similarities in host defense mechanisms between plants and animals and the importance of understanding a common evolutionary heritage. Based on the demonstrated efficacy of ABA as an immune modulatory compound and the discovery of the anti-inflammatory efficacy of the LANCL2 pathway, we screened chemical databases to identify new compounds that bind to LANCL2 and found that NSC61610 had the highest predicted binding affinity (19). Previous studies demonstrated the efficacy of NSC61610 as an anti-inflammatory compound in mouse models of colitis (19). Moreover, BT-11, a new LANCL2 ligands being developed for treating IBD, has an outstanding safety profile based on single and 14-day repeated-dose toxicology studies in rats, and it outperforms current IBD treatments in mice with dextran sodium sulfate colitis (20–22). Recently, the binding of LANCL2 to ligand has been shown to affect the cellular localization of LANCL2 resulting in multiple methods of promoting downstream effects (27).

Our *in vivo* results demonstrate for the first time that oral treatment with NSC61610 and activation of the LANCL2 pathway ameliorates pulmonary immunopathology during influenza A virus infection by suppressing inflammation and enhancing IL-10-mediated immunoregulatory responses in the lungs. Specifically, oral treatment with NSC61610 lowered infiltration of the airway mucosa and submucosa and decreased epithelial necrosis in lungs of infected mice. NSC61610 exerted its anti-inflammatory effect by suppressing TNF-α and MCP-1 expression during early and peak phases. TNF-α is a pro-inflammatory cytokine implicated in priming epithelial cells for induced cytokine and chemokine production during influenza A virus infection, while MCP1 contributes to the recruitment of immune cells (28). Anti-TNF-α humanized antibodies, such as Remicade (Centocor, Malvern, PA, USA) and Humira (Abbott Laboratories, Abbott Park, IL, USA) are biologics that have been approved by the Food and Drug Administration as therapeutics against immune-mediated diseases, such as IBD (29). The discovery of a small molecule with an oral route of administration that decreases these two inflammatory mediators expression through the selective and novel LANCL2 pathway holds similar promise in the treatment of autoimmune disorders and pathogen-initiated immunopathologies. At the cellular level, oral NSC61610 treatment significantly decreased the numbers of infiltrating neutrophils in the lungs of influenza virus-infected mice. Pulmonary neutrophil infiltration is a prominent feature of the early inflammatory response to influenza virus infection of humans, ferrets, and mice (30). Neutrophils constitute a large proportion of the inflammatory leukocytes infiltrating the lung during influenza virus infection. Although their role in influenza virus clearance is not yet well defined, it has been suggested that excessive neutrophils and neutrophil extracellular traps contribute to acute lung injury of influenza pneumonitis (31).

While differences in inflammatory markers exist throughout the course of infection, the efficacy of oral treatment with NSC61610 is most apparent during the recovery phase, in which the increased activation of immunoregulatory pathways is most prevalent. Similar improvements in the recovery phase were reported by oral treatment of influenza virus-infected mice with ABA, the first LANCL2 ligand discovered (14). Notably, the administration of ABA has been shown to activate PPARγ in a LANCL2-dependent manner suggesting that LANCL2 agonists may contribute to the activation of this regulatory pathway (11). We provide molecular evidence *in vivo* that NSC61610 functions in a LANCL2-dependent manner during the recovery phase of infection, since the beneficial effects of NSC61610 treatment observed in wild-type mice are abrogated in LANCL2 knockout mice. Indeed, the largest differences in clinical disease measures, such as weight loss and mortality occur after the peak of infection as the NSC61610-treated wild-type group experienced an earlier and more pronounced weight gain and decrease in mortality during this phase. Oral treatment with NSC61610 triggers a shift toward a regulatory tissue environment, evidenced by the increased LANCL2-dependent expression of IL-10 in the lungs.

As a crucial regulatory cytokine, IL-10 has previously been shown to suppress pulmonary inflammation and tissue damage (32, 33). The cytokine exerts its regulatory control through a signaling cascade resulting in the downregulation of inflammatory cytokines, such as MCP-1 or IFN-γ, the rampant production of which contribute to the damaging cytokine storm (34). At the cellular level, oral treatment with NSC61610 induced increased levels of IL-10-producing CD8+ T cells and CD11b+ F4/80hiCX3CR1+ macrophages in the lungs. CX3CR1+ macrophages are a myeloid cell type prominent in the promotion of a homeostatic tissue environment, predominately tied to the control of intestinal immune responses to bacteria (35). Some inflammatory subsets of macrophages have been identified as susceptible to influenza infection and are crucial mediators of the well-categorized cytokine storm associated with the influenza virus (36). The ability of LANCL2 activation to promote a regulatory, IL-10-producing, macrophage population suggests efficacy of this pathway to control the damaging effects of the influenza virus in the lungs throughout the course of infection. CD8+ T cell responses mediate resistance against intracellular infections through effector mechanisms with the potential to defend against infection (37). For example, CD8+ T cells could eliminate influenza-virus-infected targets *via* the perforin/granzyme B, Fas/FasL, or TRAIL pathways (38). Meanwhile, CD8+ T cells have previously been identified as the main producer of IL-10 in the lungs during influenza infection (33). While recent evidence suggests that the production of IL-10 from T cells may be crucial in switching from innate to adaptive immunity during infection and lesser production may be connected to enhanced morbidity in young populations (39). Treatment with NSC61610 or similar LANCL2 ligands may help to boost this switch and prevent age-associated morbidities. The production of IL-10 from this subset, which can also be driven to secrete the effector cytokine IFNγ, is initiated in part through the presence of IL-4 within the environment (40).

IL-4 is a prototypical Th2-associated cytokine that is also produced by dendritic cells and innate lymphoid cells (41, 42). In particular, the loss of the type 2 innate lymphoid cell population during influenza infection creates a loss of epithelial integrity and decreased lung function (43). The increased expression of multiple ILC2-related genes upon treatment with NSC61610 indicates the involvement of LANCL2 in the generation and maintenance of this cellular phenotype. The dual roles of IL-4, as a chemoattractant stimulant of macrophages and eosinophils and an inhibitor of pro-inflammatory cytokines, such as TNF-α and MIP-1, may suggest that its acute elevation can allow for ample recruitment of antiviral cells while decreasing the likelihood of an excessive cytokine storm (44, 45). In addition to the IL-10-related effects, a key mediator of the regulatory response is the growth factor, amphiregulin. A direct product of immune cells, amphiregulin has both traditional growth factor effects, in the maintenance and stimulation of epithelial cell growth, as well as additional regulatory mechanisms, *via* the promotion of regulatory T cells (46, 47). The increased expression of amphiregulin with LANCL2 activation may be a key component in the reduction of epithelial necrosis and lung damage during infection.

PMN-MDSCs, alternatively referred to as granulocytic MDSC, have been shown to resolve inflammation through the attenuation of T cell expansion and inflammatory cytokine production *via* direct cell-to-cell contact, producing a highly local and specific suppressive effect (48). The presence of PMN-MDSCs during the neutralization of IL-10 suggests that this cellular phenotype is capable of being established by NSC61610 in the absence of IL-10. Without the amplification of immunoregulatory effects by IL-10, the expanded MDSC population is unable to reduce disease severity or aid in the resolution of infection. This suggests that, while MDSCs may assist in the resolution of infection, IL-10-producing cells are the critical effectors of NSC61610 treatment and LANCL2 activation.

The spectrum of regulatory effects promoted by LANCL2 is dependent on signaling within myeloid cells and T cells. The promotion of IL-10-producing macrophages and CD8+ T cells is lost both in T cell and myeloid-specific knockouts of LANCL2 suggesting that interplay between the cell types is necessary to induce these effects. In contrast, the suppression of inflammatory cytokine production and increased number of alveolar macrophages remained intact within T cell knockouts of LANCL2. Alveolar macrophages are responsible for initiating many of the virus clearing responses in addition to the promotion of tissue remodeling and prevention of secondary infections (49–51). Therefore, the myeloid LANCL2- and IL10-dependent effects of NSC61610 administration are greatly beneficial to the alveolar macrophage-mediated pathways of host defense.

A concern with immunoregulatory treatment of infectious disease is impaired clearance or increased burden of the infectious agent. However, no difference in viral load was observed at day 3 post-infection with NSC61610 treatment. When also treated with Tamiflu, the viral load at day 3 post-infection was decreased. Despite a lower viral load, mice treated only with Tamiflu did not exhibit the regulatory responses exhibited by NSC61610-treated mice, suggesting the regulatory benefits of NSC61610 are independent of changes in viral load. Indeed, the therapeutic efficacy of NSC61610 has been shown in non-infectious disease models (15). Additionally, treatment with NSC61610 significantly increased survival rates compared to Tamiflu-treated mice. While Tamiflu is directly effective against specific strains of influenza A, there is risk for adaptive strains to evade its inhibitory effects. Also, the treatment schedule of Tamiflu is very dependent on fast identification and treatment initiation with as little as a 24 h delay nullifying the beneficial effects. In contrast, NSC61610 treatment was initiated at 24 post-infection in all studies and retained effects.

Though not analyzed presently, the immunoregulatory benefits of LANCL2 activation during resolution of influenza virus infection may help to bolster lasting memory responses to the viral antigen. Across viral, bacterial, and parasitic infections, the increased production of IL-10 has been shown to aid in the maturation of memory CD4+ and CD8+ T cells (52–54). As a result, treatment with NSC61610, or similar LANCL2 ligand, could also aid in the efficient generation of highly specific response upon secondary exposure. However, conflicting reports exist on the effect of IL10+ CD8+ T cells, MDSCs, and immunoregulatory therapies on the ability to expand immediate memory responses (55, 56). Therefore, further research should be conducted into the effect of NSC61610 and LANCL2 activation on the generation and maturation of memory responses.

In summary, our data demonstrates for the first time that oral NSC61610 treatment ameliorates the morbidity and mortality associated with pandemic H1N1pdm influenza virus infection by suppressing the trafficking of inflammatory tissue-damaging cells (i.e., monocytes and neutrophils) and increasing IL-10-producing CD8+ T cells and regulatory macrophages in the lungs in a LANCL2-dependent manner, thereby validating the role of the LANCL2 pathway as a novel host-targeted therapeutic against influenza that modulates the balance of effector and regulatory host responses in the lungs and systemically. NSC61610 improves clinical measures of disease compared to a current standard of care, oseltamivir (Tamiflu). Given the risk of developing resistance to Tamiflu and other agents targeting directly the virus, future studies should explore utilizing LANCL2-based host-targeted therapeutics in combination with lower doses of licensed antiviral drugs.

## Author Contributions

RH and JB-R conceived and coordinated the study. AL, NT-J, VZ-R, VG, and PL performed and analyzed the experiments described by Figures 1–8. AL, JB-R, and RH designed experiments and wrote the manuscript. SK helped in the interpretation of the SPR analyses. All authors reviewed the results and approved the final version of the manuscript.

## Conflict of Interest Statement

The authors declare that the research was conducted in the absence of any commercial or financial relationships that could be construed as a potential conflict of interest.
